# Pseudotumor in the Setting of Metal-on-Metal Total Hip Arthroplasty

**DOI:** 10.7759/cureus.8255

**Published:** 2020-05-23

**Authors:** Navraj S Sagoo, Ruhi Sharma, Connor S Johnson, Kelly Stephenson, Kessiena L Aya

**Affiliations:** 1 Orthopedic Surgery, The University of Texas Medical Branch at Galveston, Galveston, USA; 2 Orthopedic Surgery, Ross University School of Medicine, Bridgetown, BRB; 3 Orthopedic Surgery and Rehabilitation, The University of Texas Medical Branch at Galveston, Galveston, USA

**Keywords:** pseudotumor, total hip arthroplasty, metal-on-metal, metallosis

## Abstract

Metal-on-metal (MoM) hip resurfacing/replacement is a highly discussed topic in arthropathy, and the impact of its complications is still being elucidated. We report the case of a patient who presented with severe stomach pain due to a symptomatic psoas fluid collection that was later shown to communicate with a MoM total hip prosthesis. A MoM pseudotumor presenting as persistent stomach pain due to an aseptic psoas fluid collection is a rare complication. The case may support an earlier diagnosis in at-risk patients, and it outlines a suggested workup and treatment plan.

## Introduction

Total hip arthroplasties (THAs) have played an essential role in treating debilitating hip arthropathy for more than 50 years [1]. The advent of metal-on-metal (MoM) hip arthroplasty brought in a new generation of prosthetic hardware that placed emphasis on enhanced stability and durability. With their smooth articulating surfaces and potential for a significantly lower wear rate, these implants were initially thought to allow for a longer-lasting prosthesis [2,3]. Accordingly, in the 2000s, it was reported that up to 35% of THAs in the United States were comprised of MoM bearings, marking a significant acceptance in these arthroplasties [4,5]. 

In recent years, numerous complications associated with MoM THAs have come to light. Evidence now shows poor survivorship of MoM bearings, with significantly higher revision rates when compared to other hip bearings [6-8]. Additionally, the gradual detachment of metal ions, namely cobalt and chromium, may lead to a progressive “metallosis” of the periprosthetic tissues, which describes an inflammatory reaction involving local tissue necrosis, metal deposition along the periarticular tissues, and the potential formation of an aseptic, granulomatous mass known as a pseudotumor [7]. These masses often develop near MoM prostheses, can be characterized as cystic or solid, and go by a variety of terms [7].

## Case presentation

A 53-year-old man, known to have human immunodeficiency virus (HIV), presented to the emergency department with a one-week history of worsening of his chronic abdominal pain. The pain was poorly localized, but mainly in the right lower quadrant of the abdomen, with occasional radiation to the right hip and thigh and worsening upon hip flexion. He denied any changes in his bowel/bladder habits, but admitted to experiencing subjective fevers, chills, nausea, and multiple episodes of vomiting. His vital signs and complete blood count (CBC) were within normal limits, but his erythrocyte sedimentation rate (ESR) and C-reactive protein (CRP) values were elevated (ESR = 12 mm/hr, normal < 10 mm/hr; CRP = 1.6 mg/dL, normal < 0.8 mg/dL).

The patient’s medical history was significant for HIV (CD4 count 340 cells/µL), hypertension, chronic left-sided abdominal pain due to left-sided kidney stones, and a history of drug-seeking behavior. His surgical history was significant for a right MoM THA 11 years prior for symptomatic avascular necrosis, and he denied any pre- or postoperative complications. Surgery records, including the implant type, were unavailable. 

**Figure 1:**
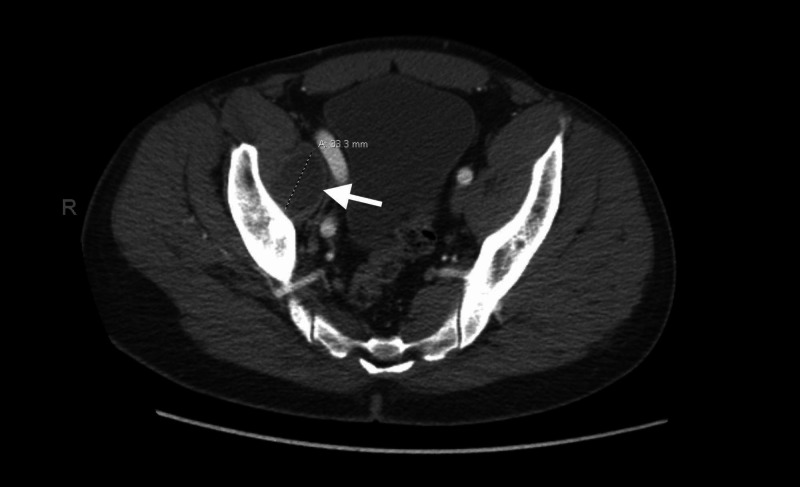
CT scan of the abdomen and pelvis. A well-circumscribed low-density collection is noted in the right psoas muscle, which measures 3.5 x 3.2 cm.

On examination, there was tenderness to palpation of the right lower quadrant, inability to fully isometrically contract the quadratus muscles, and restriction of active and passive hip flexion due to pain. Given the patient’s history of kidney stones, a CT scan with contrast of the abdomen and pelvis was obtained (Figure 1). 

This scan was significant for nonobstructive left renal stones and a well-circumscribed low-density collection in the right psoas muscle (3.5 x 3.2 cm). The stones were nonobstructive and on the contralateral side; therefore, it was unlikely that they were causing the patient’s pain. Given the likelihood of either a psoas hematoma or abscess, along with concerning neurological findings during the musculoskeletal examination, the patient was admitted and given an extensive inpatient workup. Interventional radiology (IR) was consulted, and a right iliopsoas ultrasound-guided aspiration was performed along with placement of a drain. The aspirate was noted to be viscous and bloody. Cultures of the aspirate were subsequently negative for microbes.

**Figure 2:**
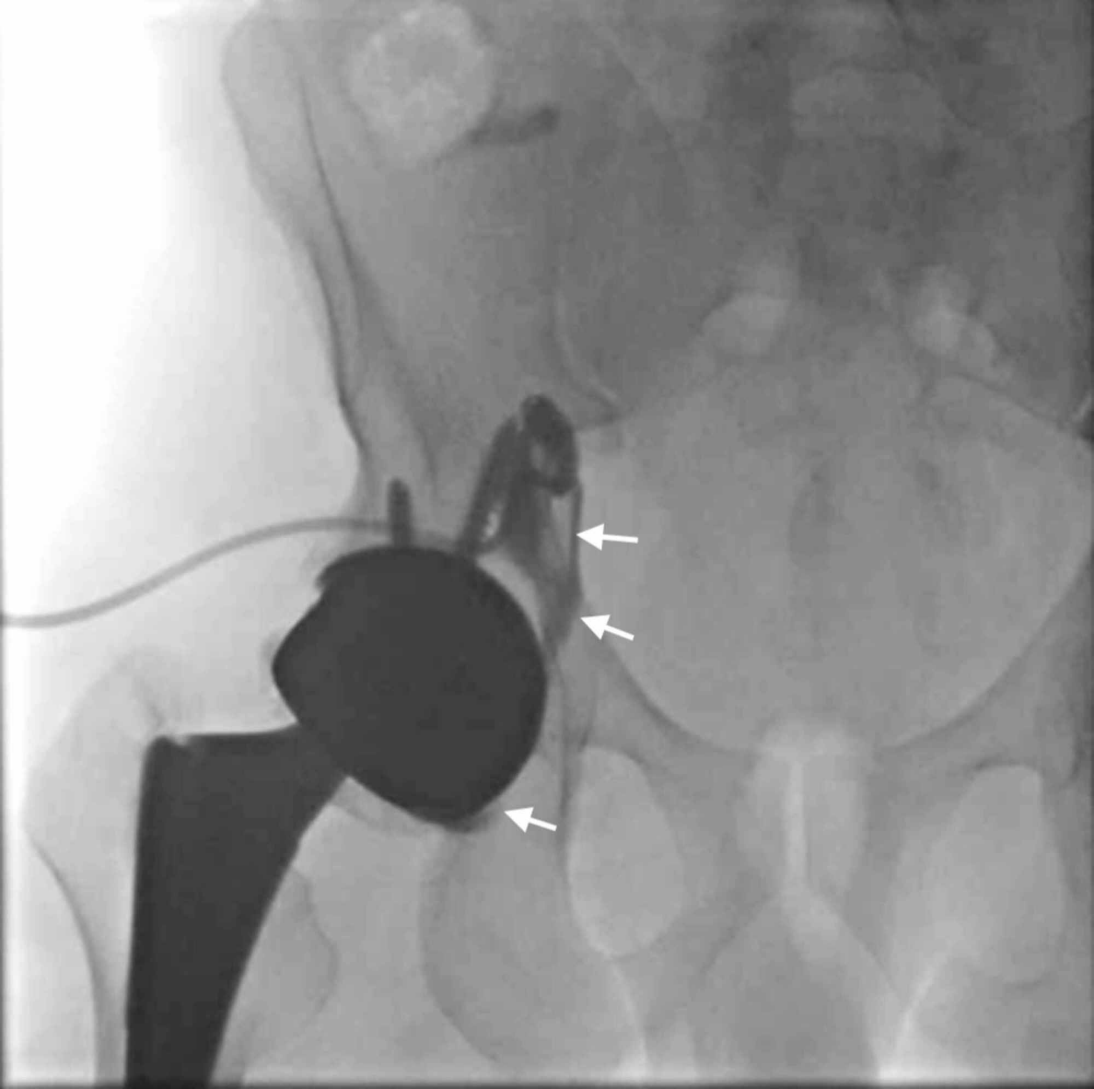
Sinogram outlining a stable cavity within the right Iliopsoas muscle. Contrast medium (white arrows) can be seen escaping into a fistula with the right hip joint space.

Due to the patient’s persistent hip pain and slow improvement after fluid aspiration, a sinogram was performed four days later via contrast injection into the drain catheter, revealing a persistent fluid collection at the right iliopsoas, with a direct fistula to the prosthetic joint, prompting a referral to the orthopedic service for further evaluation (Figure 2).

**Figure 3:**
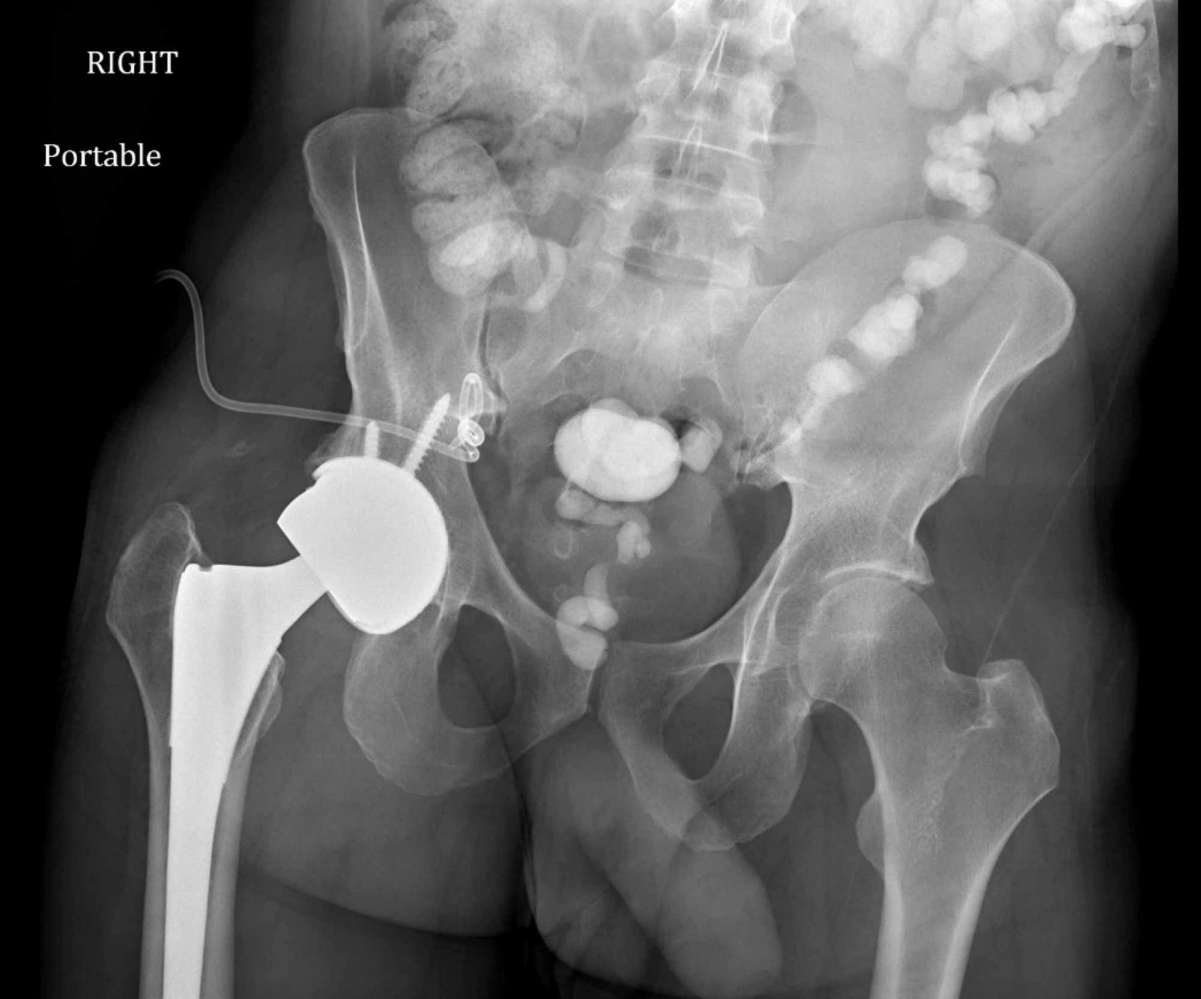
Anteroposterior view of pelvis. Hardware is in appropriate alignment without evidence of loosening. No periprosthetic fractures are seen.

Given the patient’s labs, a formal open irrigation and debridement (I&D) of the pelvis was undertaken to rule out the possibilities of a false-negative culture or an abscess that had failed IR aspiration. The fluid encountered during the I&D was black and bloody, but negative for purulence. Intraoperative fluid and tissue specimens revealed an acute inflammatory process consisting of abundant neutrophils and scattered histiocytes; however, cultures were once again negative. In addition, radiographs of the hip revealed no abnormalities (Figure 3).

Due to the consistency of the aspirate, negative cultures, normal hip radiograph, and absence of infectious clinical presentation, the possibility of a symptomatic aseptic pseudotumor was acknowledged.

A right single-stage THA revision pending an intraoperative frozen section to evaluate for periprosthetic joint infection (PJI) was planned. No gross purulence or signs of infection were noted intraoperatively; however, 20 mL of turbid trochanteric bursal fluid was aspirated, and metallosis-stained tissue was found lodged between the acetabular shell and liner and inside the synovium. The acetabular component and femoral stem were well fixed, and there was minimal corrosive wear of the trunnion.

Intraoperative tissue samples were collected from the trochanteric bursa, capsule, tissue surrounding the prosthesis trunnion, anterior capsule, and the acetabular shell membrane. Lab impressions in each sample suggested no acute inflammatory processes (0-1 neutrophils per high-power field), so the decision was made to proceed with a single-stage revision. A Smith & Nephew R3™ (London, UK) 56-mm acetabular shell, R3 0° 36-mm acetabular liner, and an Oxinium™ (Smith & Nephew, London, UK) 36-mm femoral head with a +8-mm taper were used to replace the pre-existing hardware. Appropriate alignment of the implants and leg lengths was confirmed with intra- and postoperative imaging. The remainder of his hospital course was standard with the exception of his chronic pain. He ambulated with physical therapy on postoperative day 4 and was discharged on the same day with plans to follow up with orthopedics and pain management.

**Figure 4:**
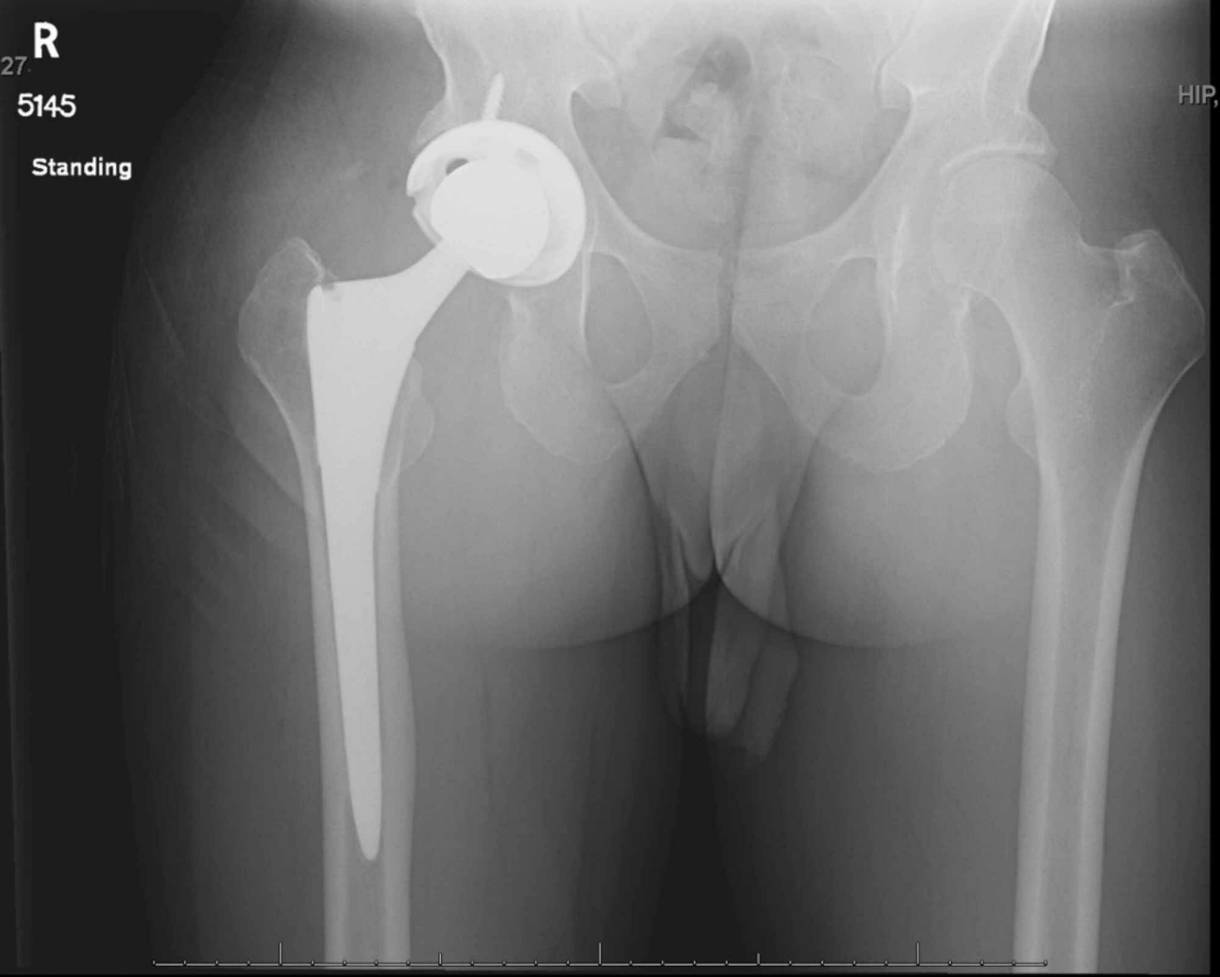
Anteroposterior view of pelvis demonstrates right hip total arthroplasty revision in appropriate alignment.

The patient had routine follow-up at two weeks and one month. At his one-month postoperative visit, the incision was well healed with no drainage or signs of infection. There was minimal pain with flexion, extension, and internal and external rotation of the leg with improvement of abdominal pain. Imaging suggested stable hardware (Figure 4). 

Since the patient’s clinical presentation and white blood cell count (5.2 x 10^3^/µL) did not indicate infection, he was instructed to continue therapy and follow up with a pain clinic. At one year postoperatively, the patient was contacted by mail and telephone and said he had not experienced any pain episodes since his last follow-up, and his charts indicated that he had not been seen for any further complications with his hip.

## Discussion

A literature search reveals multiple case reports highlighting the variable clinical manifestations of MoM THA pseudotumors. Examples include unilateral leg edema, nerve palsy, venous compression simulating a deep vein thrombosis, and proximal femur invasion [9-12]. However, this case is unique in that it presented as a symptomatic MoM pseudotumor with extension from the hip joint to the iliopsoas mass in the pelvis, resulting in acute lower abdominal pain and motor deficits.

Recent studies have demonstrated that MoM THAs have a prevalence of pseudotumor formation as high as 60% [13-15]. Given their high prevalence and diverse presentations, a large degree of clinical suspicion should be maintained. Given this patient’s complicated medical history, differential diagnoses for this case included several intrinsic (infection, instability, aseptic loosening) and extrinsic (hernia, diverticulitis, kidney stones, malignancy) causes [16]. 

Cases such as these require a systematic and efficient surgical approach to be quickly and successfully resolved. As such, a comprehensive laboratory evaluation including ESR, CRP, serum metal ion levels, CBC with differential, imaging modalities with CT scan and sinography, and a hip percutaneous aspiration were all required before reaching a definitive diagnosis after ruling out PJI with a formal I&D. Because elevated ESR and CRP are unreliable for the diagnosis of PJI in the face of metallosis from a MoM THA, formal I&D with biopsy was felt to be indicated due to the recurrence of fluid collection [17]. Serum ion levels have not been shown to have a consistent correlation with MoM prosthetic wear [13,15,18,19]. However, the patient’s elevated serum cobalt ion levels (3.3 μl/L, reference < 1.0 μl/L) were still taken into account.

Several other factors demonstrated that a revision arthroplasty was indicated. The acute nature of the pain elicited on hip flexion and the difficulty in isometrically contracting the quadriceps both potentially indicated iliopsoas dysfunction with femoral nerve involvement. Additionally, the psoas mass and prosthesis were likely interrelated since they were connected by a fistula. 

Pathological specimens were sent out to examine for infectious or inflammatory causes related to the patient’s presentation and implant failure during and after the I&D and revision arthroplasty. This characterization of intraoperative specimens is essential during a diagnostic workup, and was pivotal in diagnosing a pseudotumor. The revision arthroplasty resulted in a notable improvement from a functional standpoint. The patient was mobile postoperatively, and had improvement in his abdominal pain and muscle deficits through his final follow-up visit.

## Conclusions

This case highlights the importance of a systematic approach in diagnosing a large symptomatic pseudotumor following MoM THA. A low threshold for clinical evaluation should be maintained for early and effective management, with particular consideration given to complex patients, such as those with HIV, to rule out other possible differential diagnoses. Here, we discussed a case in which an HIV-positive man presented with acute abdominal pain, motor deficits, and a surgical history significant for a right MoM THA. A comprehensive laboratory evaluation including imaging modalities with CT scan and sinography, a hip percutaneous aspiration, and a formal I&D were all required in order to diagnose a pseudotumor. A single-stage revision arthroplasty resulted in a notable improvement of his symptoms. In cases similar to this one, a large symptomatic pseudotumor with periarticular soft-tissue destruction provides a clear indication for direct operative management. However, given their diverse clinical manifestations, further research is required to provide an informative and robust clinical guideline to follow when treating these patients. 
